# The Mysterious Ways of ErbB2/HER2 Trafficking

**DOI:** 10.3390/membranes4030424

**Published:** 2014-08-06

**Authors:** Vibeke Bertelsen, Espen Stang

**Affiliations:** Department of Pathology, Oslo University Hospital, Rikshospitalet, Post Box 4950 Nydalen, 0424 Oslo, Norway; E-Mail: vibeke.bertelsen@rr-research.no

**Keywords:** ErbB2, HER2, Hsp90, endocytosis, clathrin-coated pits, nuclear trafficking, antibodies, ubiquitination

## Abstract

The EGFR- or ErbB-family of receptor tyrosine kinases consists of EGFR/ErbB1, ErbB2/HER2, ErbB3/HER3 and ErbB4/HER4. Receptor activation and downstream signaling are generally initiated upon ligand-induced receptor homo- or heterodimerization at the plasma membrane, and endocytosis and intracellular membrane transport are crucial for regulation of the signaling outcome. Among the receptors, ErbB2 is special in several ways. Unlike the others, ErbB2 has no known ligand, but is still the favored dimerization partner. Furthermore, while the other receptors are down-regulated either constitutively or upon ligand-binding, ErbB2 is resistant to down-regulation, and also inhibits down-regulation of its partner upon heterodimerization. The reason(s) why ErbB2 is resistant to down-regulation are the subject of debate. Contrary to other ErbB-proteins, mature ErbB2 needs Hsp90 as chaperone. Several data suggest that Hsp90 is an important regulator of factors like ErbB2 stability, dimerization and/or signaling. Hsp90 inhibitors induce degradation of ErbB2, but whether Hsp90 directly makes ErbB2 endocytosis resistant is unclear. Exposure to anti-ErbB2 antibodies can also induce down-regulation of ErbB2. Down-regulation induced by Hsp90 inhibitors or antibodies does at least partly involve internalization and endosomal sorting to lysosomes for degradation, but also retrograde trafficking to the nucleus has been reported. In this review, we will discuss different molecular mechanisms suggested to be important for making ErbB2 resistant to down-regulation, and review how membrane trafficking is involved when down-regulation and/or relocalization of ErbB2 is induced.

## 1. Introduction

Receptor tyrosine kinases (RTKs) are transmembrane proteins with an extracellular ligand binding region and an intracellular tyrosine kinase domain that play important roles both in regulation of normal cells and in the development and progression of various diseases, especially cancer. Ligand binding, followed by activation of the kinase domains and initiation of signaling pathways, occur at the plasma membrane. Endocytosis and intracellular membrane trafficking are thus crucial in regulation of RTK expression and activity. ErbB2, also known as HER2, belongs to the ErbB- or epidermal growth factor receptor (EGFR)-family of RTKs. In addition to ErbB2, the ErbB-family consists of EGFR/ErbB1, ErbB3/HER3, and ErbB4/HER4. Ligand binding induces both receptor homo- and heterodimerization, but each receptor has its specialties. While EGFR, ErbB3, and ErbB4 each bind a number of different ligands, no ligand that binds to ErbB2 has been identified (reviewed in [1]). However, in contrast to the other members of the family, ErbB2 has a conformation where the dimerization arm is constitutively exposed, and ErbB2 is the preferred dimerization partner [1,2,3]. Furthermore, ErbB2 is resistant to down-regulation, and upon ligand-induced heterodimerization it also inhibits down-regulation of the dimerization partner (reviewed in [4,5]). Due to this, ErbB2 plays an important role in regulation of activity and signal transduction mediated by members of the ErbB-family.

In this article we will review and discuss reasons why ErbB2 generally is resistant to down-regulation and further describe and discuss how this resistance can be overcome. What is clear is that ErbB2 interacts with Hsp90, and that Hsp90 inhibitors cause down-regulation of ErbB2. Down-regulation of ErbB2 can also be induced upon incubation with antibodies recognizing its extracellular domain. Exactly how such down-regulations occur is, however, widely debated. ErbB2 can also be translocated to the nucleus and possible membrane transport pathways will be discussed.

## 2. ErbB2 Molecular Conformation and Interaction with Hsp90

### 2.1. The Molecular Conformation of ErbB2

**Figure 1:**
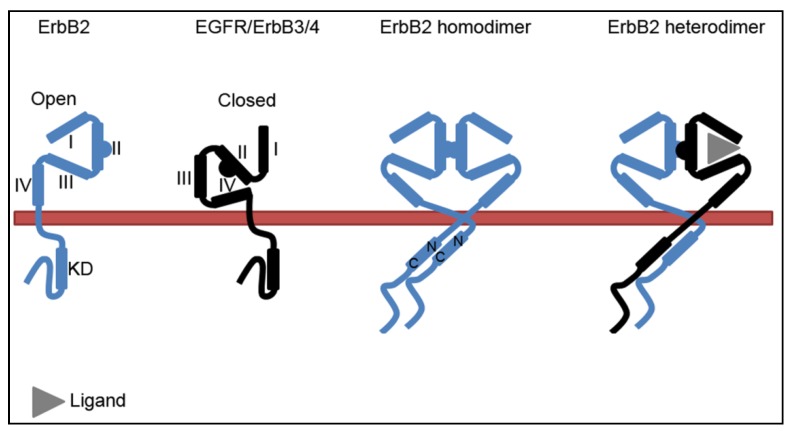
Conformation of monomeric and dimeric ErbB-proteins. While the extracellular region of ErbB2 constitutively adopts an open conformation, EGFR, ErbB3, and ErbB4 depend on ligand binding to change from a closed to an open conformation and to participate in dimerization. In dimers, the kinase domains (KDs) interact in an asymmetric fashion where the C-terminal of one KD interacts with the N-terminal of the other. I-IV refer to the respective domains in the extracellular region of the receptors.

All members of the ErbB-family share the same general composition with an N-terminal extracellular region, a transmembrane domain, and a C-terminal intracellular region (Figure 1). The extracellular region contains four domains. While domain I and III are involved in ligand-binding, domain II, also known as the dimerization arm, is important for receptor dimerization. Domain IV has an auto-inhibitory function by interacting with domain II and keeping the dimerization arm buried. Generally the ErbB-proteins depend on ligand-binding to adopt an open conformation of the extracellular region and exposure of the dimerization arm. ErbB2 does, however, have a constitutive open conformation, and this is probably the major reason why the ligand-less ErbB2 is the preferred dimerization partner [1,2,3]. The intracellular region of the receptors consists of a juxtamembrane domain, the kinase domain and a C-terminal regulatory domain. The kinase domain is further divided into N- and C-terminal lobes. Upon receptor dimerization the two intracellular domains adopt an asymmetric conformation where the N-terminus of one kinase domain interacts with the C-terminus of the other kinase domain [6].

In addition to full length ErbB2, truncated forms of ErbB2, collectively referred to as p95HER2, exist in a subgroup of ErbB2 positive cancers. p95HER2 can arise by at least two different mechanisms, protease-mediated shedding of the extracellular domain (ECD) or translation from internal translation codons (for recent reviews see [7,8]). ADAM (A Disintegrin And Metalloproteinase) 10 [9] and matrix metalloproteinases like MMP-1 [10] are reported to cleave ErbB2 within the extracellular juxtamembrane region. This results in ECD shedding and formation of a p95HER2 also referred to as 648-CTF (carboxy terminal fragment). Initiation of translation from alternative methionine residues can give rise to other p95HER2 variants known as 611-CTF and 687-CTF, where 611 and 687 refer to the position of the methionine residue [11]. Position 611 is located before the transmembrane domain whereas 687 is located in the intracellular domain. Thus, 611-CTF is a membrane protein while 687-CTF is a cytosolic protein and, as such, not directly involved in membrane trafficking. 687-CTF is, however, reported to localize to the nucleus and may thus be an important contributor to nuclear trafficking of ErbB2 (see below). Otherwise, not much is known with respect to membrane trafficking of p95HER2, but it was recently shown that at least the 611-CTF variant is an Hsp90 client and that Hsp90 inhibitors induced degradation of p95HER2 [12]. Whether this degradation involves ubiquitination and/or endocytosis, or if p95HER2 is cleaved at the plasma membrane by caspases or proteasomal activity (see below), is, to our knowledge, not known.

### 2.2. The ErbB2-Hsp90 Complex

Hsp90 is a major chaperone involved in quality control of newly synthesized proteins and has as such a large number of clients. Hsp90 is an ATP-ase which exists as an homodimer cycling between an open and a closed state depending on client and nucleotide binding (for recent reviews see [13,14]). While most client proteins are released from the Hsp90 complex upon reaching their mature conformation, certain kinases like ErbB2 and Akt remain in complex with Hsp90 upon maturation. The specificity of Hsp90 client interactions is regulated by various co-chaperones, such as Cdc37 (p50), which has been shown to act as a co-chaperone in the interaction between Hsp90 and client proteins containing kinase domains [15]. The Cdc37-Hsp90 complex is a chaperone for both RTKs and non-receptor kinases and is thus an important regulator of the kinome (reviewed in [16,17,18]). The Cdc37-Hsp90 complex interacts with the catalytic domain of the kinases. Disruption of the interaction between Cdc37 and Hsp90 [19] and siRNA-mediated depletion of Cdc37 [18,20] have both been reported to cause down-regulation of ErbB2. Whether this down-regulation involves membrane trafficking of ErbB2 remains to be elucidated. A loop within the N-lobe of the ErbB2 kinase domain has been shown to be critical for binding of Hsp90 [21,22]. It has further been proposed that Hsp90, by binding to this loop, sequesters ErbB2 homodimers and restrains both the catalytic activity of ErbB2 and its ability to form heterodimers [23]. This indicates that Hsp90 prevents uncontrolled ErbB2 activation and signaling. In support of this it was shown that an ErbB2 mutant that did not bind Hsp90 had enhanced kinase activity. The enhanced activity was shown to be Src-dependent, suggesting that Hsp90 directly or indirectly prevents Src association with ErbB2 [24].

Although the functions of Hsp90 have generally been considered to be intracellular, Hsp90 can be secreted upon environmental stress. A number of cancer cells secrete Hsp90 constitutively and cell surface-localized extracellular Hsp90 (eHsp90) is involved in regulation of cell motility and invasion as well as angiogenesis and cancer cell metastasis [25,26,27,28]. Like intracellular Hsp90, eHsp90 is in complex with Cdc37. Cdc37-eHsp90 interacts not only with ErbB2, but also with EGFR [29]. To our knowledge, whether eHsp90 has an effect on receptor stability and/or trafficking has not been investigated. However, eHsp90 has been shown to interact with the extracellular region of ErbB2 and to regulate ligand-induced ErbB2-ErbB3 heterodimerization [27,30]. Heterodimerization with ErbB2 inhibits down-regulation of ErbB3 [31] and eHsp90 might, thus, be important for regulation of ErbB3 turnover.

A number of Hsp90 inhibitors, mostly small molecules that inhibit binding of ATP, have been developed. Geldanamycin (GA) is widely used in laboratory studies, and GA analogs, such as 17-AAG and 17-DMAG, and about 15 other Hsp90 inhibitors, are at present in clinical trials [14,32]. However, as discussed in the conclusion, it remains unclear whether binding to Hsp90 directly is the factor that makes ErbB2 resistant to down-regulation. An alternative is that inhibition of Hsp90 causes recruitment of other molecular factors that drive down-regulation of ErbB2. Inhibition of Hsp90 induces a change in the chaperoning complex associated with ErbB2. Incubation with GA results in dissociation of Hsp90 accompanied by recruitment of Hsp70 [33] and the ubiquitin ligases CHIP (C-terminus of Hsc70-interacting protein) [34,35] and Cullin-5 (CUL5) [36,37]. ErbB2 then becomes ubiquitinated, and, as discussed below, this may be an important factor in the regulation of ErbB2 stability.

## 3. Why is ErbB2 Resistant to down-Regulation?

Down-regulation of receptors belonging to the ErbB-family can occur in several different ways. EGFR is one of the classic receptors used to study receptor-mediated endocytosis. Although ligand-induced internalization of EGFR originally was considered clathrin-dependent, recent studies show that clathrin-independent pathways are also involved. EGFR binds several different ligands and the internalization pathway appears to depend on which ligand is bound, concentration of the ligand, to what extent the EGFR becomes ubiquitinated, as well as the cell type [38,39]. Henriksen *et al.* [39] found that while EGF and TGF-α mainly induced clathrin-dependent internalization, HB-EGF and BTC additionally induced clathrin-independent pathway(s). Sigismund *et al.* [38] further showed that EGF can, in a concentration- and cell type-dependent manner, induce clathrin-independent EGFR internalization. At low EGF concentrations, which induce weak EGFR ubiquitination, EGFR internalization was found to be clathrin-dependent. However, at high EGF concentrations the EGFR is strongly ubiquitinated and was internalized in a clathrin-independent manner. Once internalized, EGFR is either recycled back to the plasma membrane or sorted for degradation in lysosomes. As for internalization, endosomal sorting depends on the ligand and to what extent the EGFR is phosphorylated and ubiquitinated [40]. TGF-α, which dissociates at endosomal pH, induces short-term phosphorylation and ubiquitination, and recycling of EGFR [40,41]. Other ligands like HB-EGF and BTC target all EGFR to lysosomes, while EGF targets most but not all EGFR for degradation [40]. The latter is possibly dependent on the EGF concentration and on the pathway by which EGFR is internalized [38]. ErbB3 was originally considered endocytosis-resistant, but a recent study showed that it is constitutively internalized in a clathrin-dependent manner and degraded [42]. The expression of ErbB3 is additionally regulated by a quantity control mechanism mediated by the ER-associated degradation (ERAD) pathway [43]. Down-regulation of ErbB4 is less well characterized, but ubiquitination leading to degradation can be induced both upon overexpression and ligand binding (reviewed in [4,5]).

**Figure 2:**
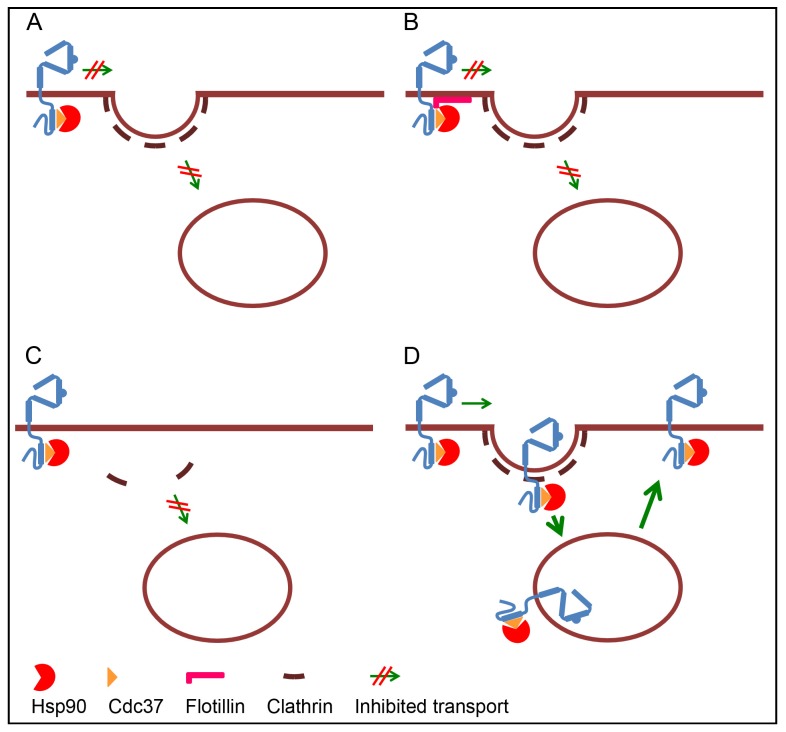
Four models for ErbB2 resistance to down-regulation. (**A**) Internalization of ErbB2 is restricted due to a lack of internalization signals, or because interaction with Cdc37-Hsp90 induces a conformation where internalization signals are hidden; (**B**) ErbB2 is retained at the plasma membrane due to interaction with proteins like flotillins and possibly other raft components; (**C**) Expression of ErbB2 inhibits the formation of clathrin-coated pits; (**D**) Internalization of ErbB2 is not inhibited, but ErbB2 in endosomes recycles rapidly back to the plasma membrane. Interaction with Hsp90 has been suggested to sequester ErbB2 homodimers, but for simplicity only monomeric ErbB2 is drawn in the figure. Different internalization pathways may be involved, but also for simplicity, only clathrin-dependent internalization is illustrated.

Localization studies demonstrate that ErbB2, apart from newly-synthesized ErbB2 in the ER/Golgi region, is restricted to the plasma membrane where it is concentrated on cellular protrusions [44]. Even in cells overexpressing ErbB2 where ErbB2 is constitutively activated, only minor amounts localize to endocytic compartments. Also the EGFR mainly localizes to the plasma membrane in resting cells. Thus, the lack of endosomal localization of ErbB2 does not, *per se*, tell anything about its endocytic capability. Furthermore, the lack of ErbB2 specific ligands makes it impossible to study ligand-induced internalization of ErbB2 homodimers. However, while EGFR is endocytosed and can be degraded upon ligand-induced homodimerization, heterodimerization with ErbB2 inhibits down-regulation of EGFR [45,46], supporting the notion that ErbB2 is resistant to down-regulation. Why the localization of ErbB2 is restricted to the plasma membrane is debated. While several studies indicate that ErbB2 is endoctytosis-resistant or -deficient due to an active retention or to the lack of internalization signals [44,46,47,48,49,50,51], other studies indicate that ErbB2 is internalized but very efficiently recycled [52,53,54]. A recent study further indicates that expression of ErbB2 itself inhibits down-regulation by having a negative effect on the formation of clathrin-coated pits [55] (see Section 3.1, Section 3.2, Section 3.3 and Section 3.4 for details and Figure 2 for illustration of different possibilities).

### 3.1. Retention of ErbB2 at the Plasma Membrane

At the plasma membrane, ErbB2 is excluded from clathrin-coated pits and instead concentrated on cellular protrusions [44]. The reason for this distribution is unclear. However, ErbB2 has been shown to be concentrated in lipid rafts [56], and on cellular protrusions it colocalizes to a high degree with the lipid raft marker GM1 [44]. ErbB2 also interacts with the lipid raft-associated oncoprotein MUC1-C [57,58]. However, disruption of rafts by cholesterol depletion did not remove ErbB2 from cellular protrusions, nor did it induce its recruitment into coated pits or internalization [44]. Likewise, although knockdown or silencing of MUC1-C inhibited an otherwise constitutive ErbB2 activation, it had no effect on the ErbB2 level [57]. It should be noted that cholesterol depletion inhibits several endocytic pathways, especially clathrin-independent pathways but to a certain degree also clathrin-dependent endocytosis. It has also been reported that EGFR-internalizing coated pits can assemble within lipid rafts [59]. Still, the data show that retention of ErbB2 at the plasma membrane does not rely on cholesterol-rich domains or interaction with MUC1-C. On the other hand, a recent study showed that interaction with raft-associated flotillins stabilizes ErbB2 at the plasma membrane. Both flotillin-1 and -2 were shown to be in complex with ErbB2 and Hsp90, and depletion of the two flotillins caused internalization and degradation of ErbB2 [51]. This suggests that interaction between ErbB2 and flotillins, either directly or through Hsp90, can serve as a retention signal. In support of a retention signal, it has been reported that inhibition of Hsp90 induces a caspase- or proteasome-mediated cleavage of the C-terminal region of ErbB2 [49,60,61], which potentially could delete a region containing a retention signal. An early study using chimeric receptors consisting of the extracellular region of EGFR and the intracellular region of ErbB2 demonstrated that the C-terminal tail of ErbB2 inhibited ligand-induced internalization of the EGFR-ErbB2 chimera [47]. Later, Shen *et al.* [62] found that resistance of ErbB2 to down-regulation relies on a specific region located between amino acids F1030 and L1075. Sequence analysis showed that ErbB2 contains 34 extra residues compared to the corresponding region in EGFR, and this region was designated as the Blocking ErbB2 Degradation (BED) domain. This supports the notion that the C-terminal region of ErbB2 contains a retention signal. However, it does not exclude that ErbB2 may be retained simply due to a lack of internalization signals in its intracellular region.

### 3.2. Lack of Internalization Signals

**Figure 3:**
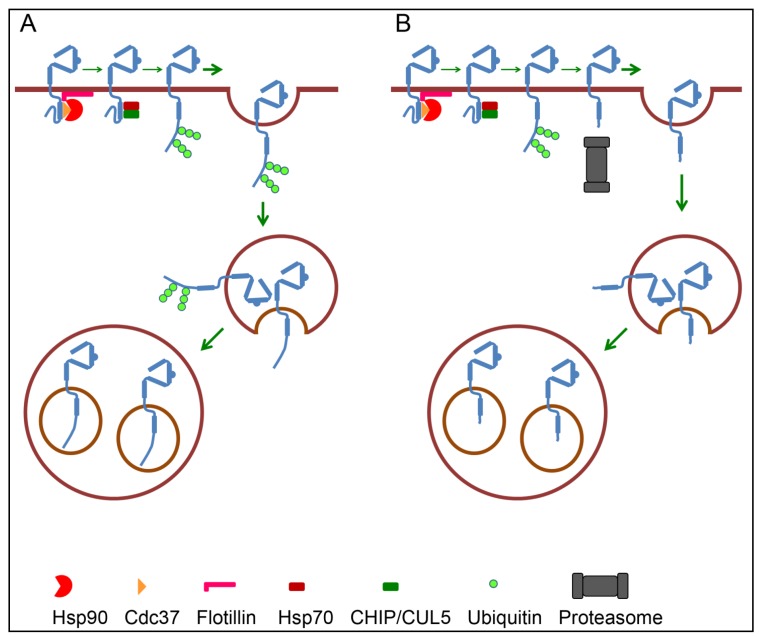
Roles for ubiquitination in ErbB2 down-regulation. Inhibition of Hsp90 causes recruitment of Hsp70 and CHIP and/or CUL5 which induce ubiquitination of ErbB2. Ubiquitination can itself serve as signal for internalization and endosomal sorting of ErbB2 (**A**), or it can induce proteasome-mediated cleavage of the intracellular domain followed by internalization and endosomal sorting of ErbB2 (**B**).

Internalization via clathrin coated pits relies on the interaction with adaptor molecules that directly or indirectly connect cargo to the clathrin lattice. A number of clathrin-associated sorting proteins (CLASPs) have been identified (reviewed in [63]). The C-terminal tail of the EGFR contains several internalization signals that collectively regulate its clathrin-mediated endocytosis [64]. When comparing the endocytosis capability of the ErbB-proteins, Baulida *et al.* [65] found that ErbB2, in contrast to EGFR, was endocytosis-impaired and did not interact with the clathrin-coated pit-localized adaptor complex AP-2. Although this does not exclude interaction with other CLASPs, it is in line with a lack of internalization signals. Alternatively, the conformation of the C-terminus may block access to internalization signals that may become exposed only upon C-terminal cleavage. The reported down-regulation of ErbB2 induced upon caspase- or proteasome-mediated cleavage of its C-terminus [49,60,61] could support this. However, since other studies demonstrate that full-length ErbB2 can be endocytosed upon inhibition of Hsp90 [48,50], cleavage is probably not obligatory and other signals must work instead or in addition. No known internalization signals have so far been identified in ErbB2. However, ErbB2 ubiquitination occurring upon inhibition of Hsp90 might be the signal needed for internalization (see Section 4.2 and Figure 3).

### 3.3. Inhibited Formation of Clathrin Coated Pits

Activation of EGFR in cells expressing low amounts of ErbB2 can induce formation of new clathrin-coated pits [66]. Over-expression of ErbB2 and formation of EGFR-ErbB2 heterodimers, do, however, inhibit EGF-induced coated pit formation [46]. This suggests that activated ErbB2 lacks the ability to induce coated pits, or even actively inhibits coated pit formation. The latter is supported by recent results showing that expression of ErbB2 caused a decrease in the number of coated pits and a general decrease in clathrin-mediated endocytosis. This indicates that over-expression of ErbB2 on its own is responsible for its low internalization rate [55].

### 3.4. Rapid Recycling

In addition to a low internalization rate, the slow turnover of ErbB2 has been explained by a rapid recycling from early endosomes [52,53,54]. However, other studies showed that treatment with monensin, an inhibitor of recycling, did not cause endosomal accumulation of ErbB2 [50,67]. Still, contrary to most other reports, Austin *et al.* [54] concluded that the Hsp90 inhibitor GA down-regulates ErbB2 by inhibited recycling and increased lysosomal sorting, and not by increased internalization. In line with this, Cortese *et al.* recently reported that GA does not have an effect on internalization of ErbB2, but instead inhibits recycling [55]. Cortese *et al.* [55] further found that GA appears to have a general effect on endosomal sorting and inhibited recycling of the Hsp90-independent transferrin receptor. Their results however contrast with the report of Austin *et al.* [54] who found that GA only had a limited effect on transferrin recycling. The reason for these discrepancies is unclear and is discussed in the conclusion (Section 7).

## 4. Down-Regulation of ErbB2 upon Inhibition of Hsp90

Hsp90 inhibitors can induce caspase- and/or proteasome-mediated cleavage of ErbB2 [48,49,60,61]. Both Lerdrup *et al.* [48] and Pedersen *et al.* [49] showed that proteasome-mediated cleavage can occur at the plasma membrane. It is, thus, possible that down-regulation of ErbB2 occurs at the plasma membrane and does not involve membrane trafficking as such. However, the original study by Tikhomirov and Carpenter [60] and numerous other studies show that down-regulation induced by Hsp90 inhibition depends on internalization of ErbB2, whether it be full-length ErbB2 or a cleaved variant. 

### 4.1. Internalization Pathways

In addition to clathrin-mediated endocytosis, a number of clathrin-independent internalization pathways exist. While one study concluded that GA-induced internalization of ErbB2 is clathrin-, dynamin- and Caveolae-independent [68], other studies conclude that the internalization is mainly clathrin-mediated [48,50,55]. The conclusion that internalization of ErbB2 is clathrin-independent was based on chlorpromazine, a drug used to inhibit clathrin-mediated endocytosis. The drug inhibited internalization of transferrin but not ErbB2. Furthermore, the authors found that expression of dominant-negative dynamin, the GTPase needed for scission of clathrin-coated pits, did not inhibit ErbB2 internalization [68]. In contrast to this, both Pedersen *et al.* [48] and Cortese *et al.* [55] found that dominant-negative dynamin indeed inhibited GA-induced internalization of ErbB2. Most importantly, Pedersen *et al.* [48] demonstrated that down-regulation of ErbB2 from the plasma membrane was almost completely inhibited by siRNA-mediated knockdown of clathrin heavy chain. Compared to treatment with chlorpromazine, knockdown of clathrin heavy chain is a more specific inhibitor. In further support of clathrin-mediated endocytosis, Lerdrup *et al.* [50] found that incubation with GA induced a redistribution of ErbB2 from plasma membrane protrusions to clathrin-coated pits. The study by Cortese *et al.* [55] showing that expression of ErbB2 had a general negative effect on formation of clathrin-coated pits also indirectly supports the role of clathrin-mediated endocytosis in control of ErbB2 internalization. Overall, we feel that current knowledge favors clathrin-mediated endocytosis as the major internalization pathway for ErbB2 upon inhibition of Hsp90. This does not exclude that alternative pathways may operate in parallel, similar to the ubiquitinated EGFR (for recent reviews see [69,70]).

### 4.2. Ubiquitination as Internalization Signal

Ubiquitination involves covalent binding of ubiquitin to lysine residues on the substrate. A substrate can become both mono- and polyubiquitinated. In the latter, several ubiquitin molecules are linked together. Each ubiquitin molecule contains seven lysine residues, and the function of a polyubiquitin chain depends on to which lysine residue the next ubiquitin molecule is linked. Ubiquitin chains linked via lysine 48, referred to as K48-linked polyubiquitination, is the classic signal for proteasome-mediated degradation. On the other hand, K63-linked chains may serve as a signal for interaction with adaptor proteins at the plasma membrane and with the endosomal sorting complex required for transport (ESCRT) machinery that sorts endosomal cargo for lysosomal degradation (for recent review see [71]). Originally, CHIP, a co-chaperone to Hsp70 and Hsp90, was identified as the ubiquitin ligase responsible for GA-induced ubiquitination of ErbB2 [34,35]. However, GA also induces ubiquitination and degradation of ErbB2 in cells where CHIP is knocked down [34]. More recently, CUL5 was identified as an alternative ubiquitin ligase for Hsp90 clients like ErbB2 [36,37]. Like CHIP, CUL5 interacts with both Hsp70 and Hsp90. In addition to ubiquitination of Hsp90 clients, recruitment of CUL5 has been suggested to cause dissociation of the Cdc37-Hsp90 complex [37]. Which ErbB2 lysine(s) becomes ubiquitinated upon inhibition of Hsp90 is still unknown, but 11 potential ubiquitination sites have been identified [72]. Hsp90 inhibition results in both K48- and K63-linked polyubiquitiation of ErbB2 [73,74]. This opens for the possibility that ubiquitination may mediate internalization of ErbB2 in at least two different ways. Ubiquitin can either itself serve as an internalization signal, or it can induce a proteasome-mediated cleavage of the intracellular region which in turn can induce internalization of ErbB2 (see Figure 3).

Supporting the role of ubiquitin as an internalization signal for ErbB2 is the finding that a chimeric ErbB2 (ErbB2-Ub), consisting of full-length ErbB2 with four ubiquitin molecules appended at its C-terminus, was constitutively endocytosed and degraded [74]. As for GA-induced internalization of wild type ErbB2, endocytosis of ErbB2-Ub was clathrin-dependent. Furthermore, over-expression of dominant-negative fragments of the ubiquitin binding CLASPs epsin1 and Eps15 negatively affected internalization of both wild type ErbB2 and ErbB2-Ub. Whether ErbB2-Ub was in a complex with Hsp90 was not directly investigated, but incubation with 17-AAG increased its turnover rate. This indicates that ErbB2-Ub was also an Hsp90 client, and suggests that ubiquitination at least partly overrules the possible retention induced by ErbB2-Hsp90 interaction.

Ubiquitination of ErbB2 has also been observed following incubation with antibodies [75,76], inhibition of proteasomal activity [73], and ligand-induced activation of an EGFR-ErbB2 chimera [77]. In contrast to the CHIP- and/or CUL5-mediated ubiquitination occurring upon inhibition of Hsp90, these ubiquitination events depend on c-Cbl, the ubiquitin ligase responsible for ligand-induced ubiquitination of EGFR [73,76,77]. c-Cbl binds to phosphorylated tyrosine 1112 (pY1112) of ErbB2, which corresponds to pY1045 of EGFR [76,77] (see also Section 4.3 and Section 5).

### 4.3. Endosomal Sorting of ErbB2

Upon internalization induced by Hsp90 inhibitors, ErbB2 initially localizes to early endosomes that also contain endocytosed transferrin. ErbB2 is then sorted to intraluminal vesicles (ILVs) of multivesicular late endosomes (multivesicular bodies = MVBs) [48,50]. Sorting of the EGFR to ILVs is regulated through interaction with the ESCRT machinery where ubiquitinated EGFR initially binds to and phosphorylates the ESCRT-0 component Hrs. Further sorting depends on the ESCRT-I, -II, and -III complexes and deubiquitinating enzymes like AMSH and UBPY/USP8. However, little is known about the role of the ESCRT machinery in endosomal sorting of ErbB2. As mentioned above, EGFR-ErbB2 chimeras consisting of the extracellular and transmembrane domains of EGFR and the intracellular region of ErbB2 can bind to and be ubiquitinated by c-Cbl [77,78]. In agreement with previous studies, the EGFR-ErbB2 chimera showed decreased ligand-induced down-regulation when compared to wild type EGFR. However, while Sorkin *et al.* [47] found that this was due to a low internalization rate, Meijer *et al.* [78] concluded it was due to an increased recycling. The increased recycling was then explained by impaired phosphorylation of Hrs and impaired deubiquitination by AMSH.

As for the EGF-induced internalization and sorting of EGFR, GA-induced internalization and sorting of ErbB2 to ILVs depend on proteasomal activity [41,48,50]. While Lerdrup *et al.* [50] found that proteasomal activity was needed for internalization from the plasma membrane, Pedersen *et al.* [48] found that proteasomal activity was needed for the formation of ErbB2-positive MVBs. Similar to what has been shown for the EGFR [41], it was found that ErbB2 accumulated on the limiting membrane of enlarged early endosomes when proteasomal activity was inhibited [48]. Since the N- and the C-terminal domains of both EGFR and ErbB2 have been shown to be intact upon sorting to ILVs [41,48,50], the receptors do not seem to be the direct target for the proteasomes, and the exact role of the proteasomal activity remains to be identified.

## 5. Effects of Anti-ErbB2 Antibodies on ErbB2 Trafficking

Antibodies have become an important tool in cancer treatment, and several potentially therapeutic anti-ErbB2 antibodies have been developed. Thus far, two humanized monoclonal anti ErbB2 antibodies, Trastuzumab (Herceptin) and Pertuzumab (Perjeta), have been approved for clinical use. Trastuzumab, which is derived from the murine antibody 4D5, binds to domain IV of ErbB2 and has been reported to prevent ligand-independent activation of ErbB2-ErbB3 heterodimers [79]. Pertuzumab is derived from the murine antibody 2C4. It binds to domain II, the dimerization arm of ErbB2, and inhibits ErbB2 heterodimerization [80,81]. Furthermore, Trastuzumab, but not Pertuzumab (2C4), inhibits shedding of the ErbB2 ECD and prevents the formation of p95HER2 [82].

Originally it was reported that Trastuzumab, or 4D5, induced internalization and degradation of ErbB2 [83,84,85]. However, later studies have concluded that Trastuzumab alone does not, or only to a very limited extent, induce internalization of ErbB2 [54,67,86,87,88]. Pertuzumab also down-regulates ErbB2, but like Trastuzumab, only to a limited extent [87,88]. One possible reason for the conflicting results is that internalization may depend on antibody-induced ErbB2 clustering. In line with this, a biotin-avidin/streptavidin-induced cross-linking of bound Trastuzumab, probably causing clustering of Trastuzumab-ErbB2 complexes, caused efficient internalization of ErbB2 [86]. Another way to induce receptor clustering is to combine two or more non-competitive antibodies. Friedman *et al.* [89] showed that Trastuzumab combined with L26, an antibody that, similar to Pertuzumab, inhibits ErbB2 heterodimerization, very efficiently down-regulated ErbB2. As a follow up, Ben-Kasus *et al.* [75] tested different combinations of anti-ErbB2 monoclonal antibodies. They found that the combination of an antibody (L431) recognizing the dimerization arm with a non-competitive, non-inhibitory anti-ErbB2 antibody, had a synergistic effect on ErbB2 down-regulation. In addition, the combination of Trastuzumab and Pertuzumab has been shown to increase the internalization and degradation of ErbB2, although in a cell-type dependent manner [87,88]. While 17-AAG- or GA-induced internalization of ErbB2 is considered clathrin-dependent, the molecular mechanisms regulating antibody-induced down-regulation have not been determined. The study by Friedman *et al.* [89] confirmed that the down-regulation involves endocytosis and that it depends on dynamin. Moreover, after incubation with colloidal gold particles coated with anti-ErbB2 antibody that probably cross-links several ErbB2 molecules, ErbB2 was found in coated pits at the plasma membrane and in endosomes [90]. An extensive cross-linking induced by incubation with an anti-ErbB2 antibody followed by colloidal gold coated with secondary antibodies, also caused clustering and internalization of ErbB2. At the plasma membrane, ErbB2 was then found both outside and within coated pits [44]. This suggests that clathrin-mediated endocytosis is involved, but does not rule out other mechanisms. A similar antibody-induced clustering of EGFR in complex with the therapeutic anti-EGFR antibody Cetuximab, showed partial localization of complexes to coated pits, but the internalization was demonstrated to be clathrin-independent macropinocytosis [91]. It has been shown that antibodies alone and/or in combination can induce ubiquitination of ErbB2 [75,76], but studies by Friedman *et al*. [89] and by Maier *et al.* [90] showed that antibody-induced down-regulation was independent of the cytoplasmic region of ErbB2. The latter indicates that ubiquitination is not needed for internalization and/or lysosomal sorting of the antibody-ErbB2 complexes. This opens for alternative pathways when it comes to antibody-induced down-regulation of ErbB2, and identification of the exact molecular mechanisms remains to be determined.

*In vivo*, it has been reported that Trastuzumab preferentially has an effect on cells expressing ErbB2 homodimers [92]. Bivalent binding of Trastuzumab may induce an orientation of the ErbB2 kinase domains such that they no longer achieve the active conformation [93]. Otherwise, an important *in vivo* effect of therapeutic antibodies is activation of immune effector mechanisms such as antibody-dependent cellular cytotoxicity, and complement-mediated cytotoxicity (for recent review see [94]).

## 6. Nuclear Trafficking of ErbB2

Despite functioning as receptors at the plasma membrane, the transmembrane RTKs have also been shown to localize in the nucleus. Since several such studies are based on subcellular fractionation, precautions should be taken with respect to whether the receptors are in the nuclear envelope or free in the nucleoplasma. However, accumulating evidence indicates a real translocation of receptor fragments and intact receptors to the nucleoplasma, where they can function both as transcriptional co-activators and as kinases (reviewed in [95,96]). The molecular mechanisms regulating nuclear localization of ErbB proteins are probably best understood for ErbB4. The translocation is initiated by ADAM 17-mediated cleavage of the ErbB4 ECD, followed by γ-secretase-mediated cleavage within the transmembrane domain. The γ-secretase-mediated cleavage results in a soluble s80 intracellular domain (ICD) fragment, which upon interaction with chaperones can be transported through nuclear pores [95]. Since the ADAM 17- and γ-secretase-mediated cleavages can occur at the plasma membrane, translocation of the ErbB4 ICD to the nucleus does not necessarily involve membrane trafficking. However, nuclear localization of ErbB3 has been reported to involve macropinocytosis [97]. The EGFR has been shown to localize to the nucleus as a short alternative splicing variant [98], as a soluble ICD formed upon ligand-induced cleavage mediated by rhomboid intramembrane proteases [99], and as an intact receptor (reviewed in [95,96]). Translocation of the intact EGFR has been shown both upon ligand binding [100,101] and upon interaction with the anti-EGFR antibody Cetuximab [102]. This translocation involves several retrograde membrane transport events. It is initiated by clathrin-mediated endocytosis [103] followed by an alternative sorting in early endosomes. Instead of recycling to the plasma membrane or sorting to lysosomes for degradation, receptors destined for the nucleus are transported to the Golgi apparatus. The molecular mechanisms controlling retrograde transport of the EGFR to the Golgi are not fully characterized, but several possible pathways exist (reviewed in [104]). Once in the Golgi, the EGFR follows a COP I-mediated retrograde transport to the ER [105]. Further translocation into the nucleus depends on importin β and the Sec61 translocon. Importin β facilitates nuclear import of the EGFR [106], probably through interaction with nuclear localization signals (NLS) located in the EGFR juxtamembrane region [107]. At what stage Sec61 becomes involved is a matter of debate. While Liao and Carpenter reported that Sec61 mediates retrotranslocation of the EGFR into the cytosol prior to interaction with importin β [100], Wang *et al.* [108] reported that Sec61 interacts with the EGFR after importin β-mediated translocation to the inner nuclear membrane. Nuclear localization has also been reported for both truncated and intact ErbB2. Alternative initiation of translation has been reported to give rise to ErbB2 CTFs that localize to the nucleus in a kinase activity-dependent manner [11]. The EGFR and ErbB2 tyrosine kinase inhibitor Lapatinib has been reported to induce a proteasome-mediated cleavage of the intracellular domain of ErbB2, resulting in a tyrosine-phosphorylated cytoplasmic p95L ErbB2 that can translocate to the nucleus [109]. However, this finding is controversial since other studies conclude that incubation with Lapatinib either causes a general stabilization of ErbB2 [110], or a shedding of the ErbB2 ECD [111]. Kim *et al.* [112] found that Lapatinib actually inhibited nuclear translocation of both EGFR and ErbB2. Translocation of C-terminal fragments of ErbB2 to the nucleus does not necessarily involve membrane trafficking. However, translocation of intact ErbB2 has been reported and appears to follow the same retrograde transport route as the EGFR, followed by importin β-mediated import and Sec61β association in the inner nuclear membrane [113,114,115]. Most studies indicate that nuclear localization of intact ErbB2 is constitutive, but a recent study showed that the progesterone receptor can transactivate and induce nuclear translocation of ErbB2 [116]. To what extent internalization induced by Hsp90 inhibitors results in nuclear localization of ErbB2 is, to our knowledge, not known.

## 7. Conclusions

The reason why ErbB2 is resistant to down-regulation remains unclear, and several discrepancies are reported. There can be many reasons for these discrepancies. Most studies referred to in this review are performed using cell lines which certainly vary with respect to expression of ErbB2 and other ErbB proteins. ErbB2 is the preferred dimerization partner and the extent of ErbB2 homo- *versus* heterodimerization will vary depending on to what extent EGFR, ErbB3 and ErbB4 is expressed. Although heterodimerization with ErbB2 has a negative effect on ligand-induced internalization of EGFR and ErbB3, it is likely that ErbB2 stability also will vary depending on the expression of these receptors. The ErbB2 expression level is also most likely a limiting factor in antibody-induced trafficking of ErbB2. The extent of antibody induced cross-linking will depend both on the density of ErbB2 at the plasma membrane, and on the avidity of the antibodies. Discrepancies are probably also due to the use of different methods and/or different antibodies used to detect trafficking and/or down-regulation of ErbB2. Both the extra- and intracellular regions of ErbB2 can be cleaved. It is, thus, important that the antigenic epitope recognized by the antibody is taken into consideration when interpreting the results. Additionally, the discrepancies probably also reflect that there are not only one, but several reasons for ErbB2’s resistance to down-regulation.

Studies using Hsp90 inhibitors clearly show that interaction with Hsp90, and most likely Cdc37, stabilizes ErbB2. However, it is still unclear whether this interaction is the main mechanism that makes ErbB2 resistant to down-regulation. One possibility is that the interaction between ErbB2 and flotillin-1 and/or -2, directly, or through Hsp90, physically retains ErbB2 at the plasma membrane. Alternatively the chaperoning effect of Hsp90 may keep ErbB2 in a conformation in which non-identified signals for internalization or endosomal sorting are hidden. On inhibition of Hsp90, such signals may be exposed as a result of a conformational change, or due to ubiquitination induced by the recruitment of Hsp70 and CHIP and/or CUL5. Such ubiquitination can serve as a signal both for internalization and for endosomal sorting towards lysosomes. Through interaction with the ESCRT machinery, ubiquitination will also serve as an inhibitor of the rapid recycling otherwise suggested for endocytosed ErbB2 [52,53,54]. Alternatively, ubiquitination may serve as a signal for proteasome-mediated cleavage of the C-terminal region of ErbB2 and in that way contribute to the exposure of otherwise hidden internalization signals. One explanation does not necessarily rule out the other. Upon overexpression, ErbB2 forms homodimers and possibly also homo-oligomers. The observation that the C-terminal tail of ErbB2 can be intact upon sorting into MVBs does not exclude the possibility that the other ErbB2 within the dimer is cleaved. 

It is suggested that multiple mechanisms collectively regulate endocytosis of the EGFR [64]. This may also be the case for other receptors like ErbB2. A fascinating aspect is the suggestion that ErbB2 directly regulates the formation of coated pits [55]. In addition to inhibited internalization, rapid recycling may be an important factor in restricting down-regulation of ErbB2. However, as pointed out in Section 3.4, the rapid recycling is controversial. There are several possible reasons for the discrepancies reported. Since ErbB does not have a ligand, its internalization is difficult to study. The conclusion that ErbB2 is endocytosis-resistant or -deficient is thus partly based on its steady state distribution with no detectable localization in endosomes even upon incubation with an inhibitor of recycling [50,67]. It is also based on its lack of known internalization signals and lack of interaction with AP-2 [65]. Finally, the identification of a C-terminal BED domain that inhibits internalization of EGFR-ErbB2 chimeras [47,62] favors the inhibited internalization model. The studies concluding that ErbB2 is rapidly recycled are based on trafficking of antibody-ErbB2 complexes [54], on ligand-induced trafficking of either EGFR-ErbB2 dimers [52,53] or an EGFR-ErbB2 chimera [77,78]. As described in Section 4.2 and Section 4.3, incubation with antibodies or ligand-induced activation of the EGFR-ErbB2 chimera may induce c-Cbl mediated ubiquitination of ErbB2. It is, thus, possible that the reported internalization and rapid recycling do not represent steady state distribution of ErbB2, but are rather due to a transient c-Cbl mediated ubiquitination. In support of this Meijer *et al.* [78] found that the EGFR-ErbB2 chimera behaved like TGF-α activated EGFR; it was ubiquitinated but rapidly recycled due to lack of interaction with ESCRT complexes.

A conceptual question that remains is what role Hsp90 plays in inhibiting down-regulation of ErbB2. The original study by Sorkin *et al.* [47] using EGFR-ErbB2 chimeras and the identification of a ErbB2 BED domain by Shen *et al.* [62] indicate that endocytosis resistance depends on a region C-terminal to the kinase domain. Since the Cdc37-Hsp90 complex binds to the kinase domain of ErbB2, interaction with Cdc37-Hsp90 is probably not the direct, or at least not the only, reason why ErbB2 is endocytosis-impaired. In support of this, several studies show that ErbB2 mutants that do not bind Hsp90 are stable [21,22,33]. This indicates that loss of Hsp90 interaction does not *per se* cause ErbB2 degradation. Furthermore, internalization and degradation of EGFR or ErbB3 is inhibited upon ligand-induced heterodimerization with ErbB2 [31,46,117]. Since Citri *et al.* [23] found that Hsp90 dissociates from ErbB2 upon heterodimerization, Hsp90 is most likely not the factor making ErbB2 heterodimers resistant to down-regulation. A recent study by Kancha *et al.* [118] also challenged the concept that interaction with Hsp90 makes ErbB2 endocytosis-resistant and/or inhibits down-regulation of ErbB2. Using different kinase inhibitors they found that the Cdc37-Hsp90 complex preferentially bound to the active conformation of ErbB2. Lapatinib, which binds to the inactive conformation of the ErbB2 kinase domain, inhibited binding of Cdc37-Hsp90 to the same extent as GA and 17-AAG, but did not induce degradation of ErbB2. However, what GA and 17-AAG but not Lapatinib induced, was recruitment of CHIP and ubiquitination of ErbB2. If this is correct, it suggests that inhibited down-regulation of ErbB2 is mainly due to an Hsp90-independent retention or to the lack of internalization and/or lysosomal sorting signals. Furthermore, it indicates that an important effect of Hsp90 inhibitors is not necessarily the dissociation of Hsp90, but the recruitment of Hsp70-CHIP and/or -CUL5 followed by ubiquitination. Ubiquitination can then serve as a signal for proteasome-mediated cleavage and/or internalization and degradation of ErbB2.

## Abbreviations

BED: Blocking ErbB2 Degradation
CHIP: C-terminus of Hsc70-interacting protein
CLASP: Clathrin-associated sorting protein
CTF: Carboxyterminal fragment
ECD: Extracellular domain
EGFR: Epidermal growth factor receptor
ESCRT: Endosomal sorting complex required for transport
GA: Geldanamycin
ICD: Intracellular domain
ILV: Intraluminal vesicle
MVB: Multivesicular body
RTK: Receptor tyrosine kinase
